# Editorial: Targeting different programmed cell death processes as therapeutic modalities for thyroid cancer

**DOI:** 10.3389/fendo.2023.1255259

**Published:** 2023-07-27

**Authors:** Sadegh Rajabi

**Affiliations:** Traditional Medicine and Materia Medica Research Center, Shahid Beheshti University of Medical Sciences, Tehran, Iran

**Keywords:** thyroid cancer, targeted therapy, programmed cell death, treatment, prognosis

It is my great pleasure to introduce this Research Topic entitled “*Targeting Different Programmed Cell Death Processes as Therapeutic Modalities for Thyroid Cancer*” that specifically highlights some of the ways to target programmed cell deaths for thyroid cancer therapy. The studies in this topic also introduce some genetic and epigenetic markers for thyroid cancer prognosis and prediction.

Ferroptosis, as a novel form of programmed cell death (PCD), has been shown to have association with thyroid cancer (1). Ren et al. conducted a study that included 497 thyroid cancer RNA expression datasets derived from the cancer genome atlas (TCGA) cohort and constructed a prognostic risk model for eight ferroptosis-related genes using Lasso-Cox regression. Indeed, they aimed to evaluate relationship between these genes and prognosis and immune cell infiltration.

Immune checkpoint inhibitors are monoclonal antibodies with anticancer ability to increase overall survival in cancer patients (2). The important contribution of tyrosine kinase inhibitors (TKIs) in the treatment of TC is highlighted in a comprehensive review by Chera et al., in which immune-related adverse events (irAEs) during the thyroid cancer therapy with TKIs have been discussed. Then, the ways to manage these adverse effects have been described.

According to the literature, X-linked inhibitor of apoptosis (XIAP) overexpression and BRAF^V600E^ mutation are associated with an aggressive phenotype of papillary thyroid carcinoma (PTC) (3, 4). Parvathareddy et al. conducted an investigation on 1600 PTC tumors to assess the prevalence of XIAP expression in combination with the BRAF^V600E^ mutation in these Middle Eastern patients and weight their prognostic value to predict disease-free survival.

Pesticide exposure is shown as one of the important factors that may induce epigenetic alterations in different neoplastic transformations (5). Salimi et al. focus on the effect of organochlorine pesticides (OCPs) on promoter methylation of three tumor-suppressor genes and four histone modifications in thyroid nodules from 61 PTC and 70 benign thyroid nodules (BTN) patients.

I have enjoyed hosting this exciting Research Topic and I thank all the authors for their excellent contributions.

## Author contributions

The author confirms being the sole contributor of this work and has approved it for publication.
